# Embryo-endometrial interaction associated with the location of the embryo during the mobility phase in mares

**DOI:** 10.1038/s41598-024-53578-z

**Published:** 2024-02-07

**Authors:** Thadeu de Castro, Machteld van Heule, Rafael R. Domingues, Julio C. F. Jacob, Peter F. Daels, Stuart A. Meyers, Alan J. Conley, Pouya Dini

**Affiliations:** 1 Department of Population Health and Reproduction, School of Veterinary Medicine, University of California, Davis, CA 95616 USA; 2 Department of Morphology, Imaging, Orthopedics, Rehabilitation and Nutrition, Faculty of Veterinary Medicine, University of Ghent, 9820 Merelbeke, Belgium; 3 Department of Animal and Dairy Sciences, The Ohio State University, Columbus, OH 43210 USA; 4 Departmento de Reprodução E Avalição Animal, Universidade Federal Rural Do Rio de Janeiro, Seropédica, Rio de Janiro 23897-000 Brazil; 5 Department of Anatomy, Physiology and Cell Biology, School of Veterinary Medicine, University of California, Davis, CA 95616 USA

**Keywords:** Embryology, Intrauterine growth

## Abstract

Embryo-maternal crosstalk is essential to establish pregnancy, with the equine embryo moving throughout the uterus on days 9–15 (ovulation = day 0) as part of this interaction. We hypothesized that the presence of a mobile embryo induces local changes in the gene expression of the endometrium. On Day 12, the endometrial transcripts were compared among three groups: uterine horn with an embryo (P+, n = 7), without an embryo (P−, n = 7) in pregnant mares, and both uterine horns of nonbred mares (NB, n = 6). We identified 1,101 differentially expressed genes (DEGs) between P+ vs. NB and 1,229 DEGs between P− vs. NB. The genes upregulated in both P+ and P− relative to NB were involved in growth factor pathway and fatty acid activation, while downregulated genes were associated with oxytocin signaling pathway and estrogen receptor signaling. Comparing the transcriptome of P+ to that of P−, we found 59 DEGs, of which 30 genes had a higher expression in P+. These genes are associated with regulating vascular growth factors and the immune system, all known to be essential in early pregnancy. Overall, this study suggests that the mobile embryo influences the endometrial gene expression locally.

## Introduction

In horses, the corpus luteum (CL) forms after ovulation and secretes progesterone (P4), which prevents the mare from returning to estrus^1^. At around day 13 of the cycle in nonpregnant mares (D0 = day of ovulation), prostaglandin F2α (PGF2α) is released from the endometrium, which induces luteolysis (P4 < 0.1 ng/mL). However, in pregnant mares, the synthesis and secretion of PGF2α are partially blocked, preventing luteolysis and thus encouraging the continued production of P4^2^. This provides receptive uterine conditions that allow the embryo/conceptus to thrive^3^. The continuous supply of progestins during pregnancy causes myometrial quiescence and is essential for embryo survival^4^. After fertilization, the equine embryo passes from the oviduct into the uterine horn at about D6^5^. The intrauterine equine embryo is first detectable by conventional ultrasonic imaging around D9 or D10^6^, and thereafter its migration can be imaged throughout the uterine lumen^7^. On D11–D12, the embryo reaches its maximum mobility in association with maximal uterine contractility^8^. Embryo mobility ceases at the time of fixation around D15–D16^9^.

During early pregnancy, the conceptus produces and sends signals to the dam and, subsequently, ensures the maintenance of the lifespan of the CL and the production of progesterone^10^. The mechanisms through which the early embryo interacts with the maternal system to prevent luteolysis vary among different species^11^. In domestic ruminants, the conceptus secretes interferons that block the endometrial expression of the estrogen receptor (ESR1) and oxytocin receptor (OXTR), which inhibits the PGF2α synthesis pathway^12^. In pigs, the anti-luteolytic mechanism was believed to involve estrogen derived from the conceptus^13^, though a more recent study indicates this is not the case^14^. Horses certainly remain one of the few domestic species in which the embryonic signals that prevent luteolysis have not yet been completely identified^15^. It is known that embryo mobility is important for the maintenance of CL activity because restriction of embryo mobility by uterine ligation leads to the loss of pregnancy^16^. Yet, the underlying mechanism involved in this intrauterine migration and embryo-maternal communication is not fully understood. In a recent study^17^, endometrial gene expression of prostaglandin E2 synthase (*PTGES*) was found to be greater in the uterine horn with the embryo than in the horn without the embryo. Additionally, the uterine horn that contained the embryo had a greater vascularity score compared to the opposite horn on days 12 and 15, noticeable within 7 min of the embryo entering the horn^18^. Prevention of embryo migration by surgical ligation of both uterine horns increased uterine tone and contractility of the horn adjacent to the embryo, while having no effect on the contralateral side that was not exposed to the embryo^6^. Furthermore, contractions of the uterine body decreased after the embryo left the uterine body^19^. These studies suggest the presence of a local intrauterine effect of the mobile embryo on the uterus, emphasizing the importance of the location of the embryo in analyses of gene expression in the endometrium.

Several studies investigated the transcriptomic profile of the endometrium to gain further insight into the molecular events underlying embryo-maternal interactions during early pregnancy in mares^20–28^. The differentially expressed genes (DEGs) between pregnant and nonbred mares during early pregnancy were mainly associated with pathways related to the immune system, angiogenesis^21^, cell-to-cell interaction^26^, and prostaglandin regulation^23^. These studies advanced our understanding of the molecular pathway associated with embryo-maternal interaction in mares. However, to our knowledge, no study investigated the global equine endometrial transcriptome considering the location of the mobile embryo in pregnant horses in comparison to nonbred mares. Thus, the present study was performed to characterize the gene expression of the endometrium of mares based on the location of the mobile embryo and to compare the gene expression in the endometrium between the uterine horns of pregnant and nonbred mares on D12 post-ovulation. We hypothesized that the presence of a mobile embryo induces local changes in the gene expression of the endometrium.

## Results

One mare in the pregnant group was omitted from the analyses due to the high duplication level (96%) of its reads. The number of mares remaining in the pregnancy and nonbred groups were 7 and 6, respectively. On average, 37 ± 0.5 million raw reads were generated from each sample, with quality over Q30 Phred score (Supplementary Table 1). Data were mapped to the equine reference genome (EquCab3.0), with an average mapping rate of 94% (range: 88.3 – 95.7%; Supplementary Table 1). Among all genes, 93.35% were categorized as protein-coding genes.

### Transcriptome profile of endometrium among the groups

**Figure 1:**
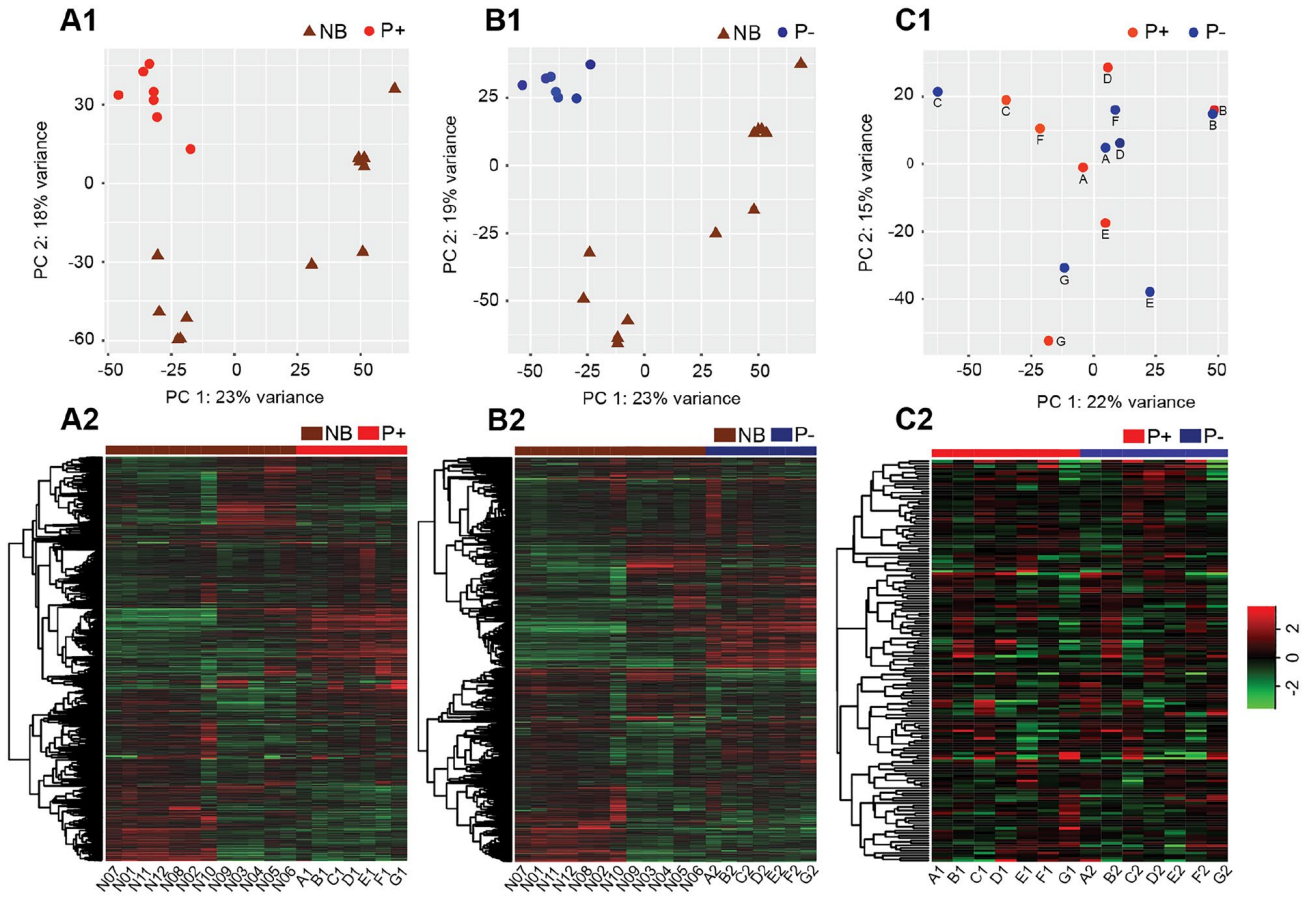
Principal component analysis (PCA) and heatmap. (**A1**) PCA and (**A2**) heatmap in P+ vs. NB; (**B1**) PCA and (**B2**) heatmap in P− vs. NB; (**C1**) PCA and (**C2**) heatmap in P+ vs. P−. There is a clear clustering between pregnant (P+ and P−) and NB groups. P+, uterine horn with the embryo; P−, uterine horn without the embryo; NB, nonbred. Each letter (A-G) in PCA and Heatmap corresponds to each mare. Graphs were generated using the ggplot2 package in R.

In the nonbred group (NB), there was no difference in gene expression between the uterine horn ipsilateral and contralateral to the CL, indicating that the sampling method, sampling order, and location of the CL did not have an influence on the gene expression pattern of the endometrium. The principal component analysis (PCA) and heatmap of the endometrium transcriptome are shown (Fig. 1A1,A2,B1,B2). The results demonstrated that samples clustered based on the group (P+, P−, and NB), and the P+ and P− samples grouped more closely together than the NB samples. The visualization of the endometrial samples of the pregnant mare (P+ vs. P−) revealed distinct clusters based on the individual mare rather than the groups (P+ and P−; Fig. 1C1,C2).

### Dynamics of gene expression between pregnant and non-bred endometrium

**Figure 2:**
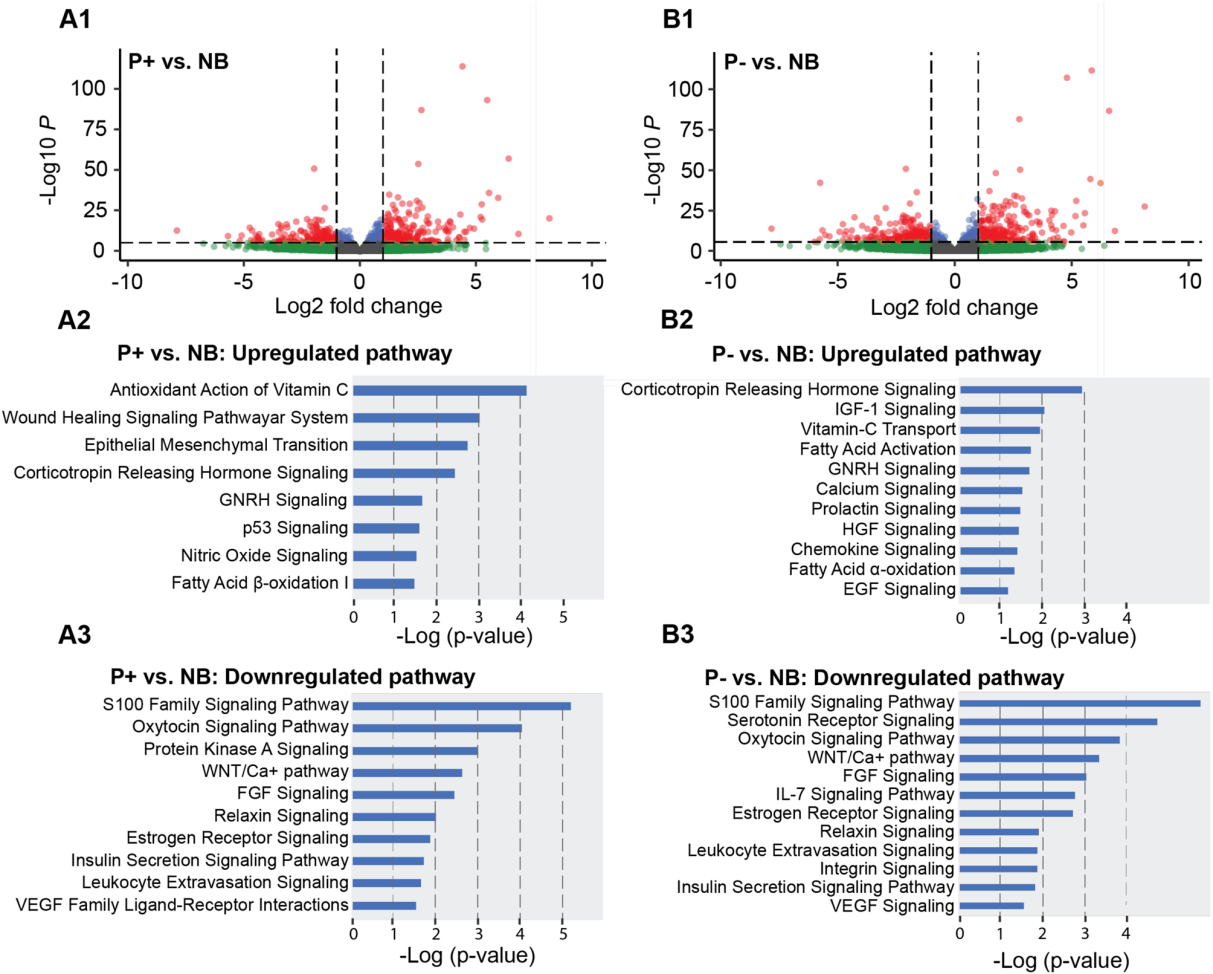
Volcano plot and the most significant upregulated and downregulated canonical pathways in P+ vs. NB and P− vs. NB. The red dots represent the DEGs genes with adjusted p-value < 0.05 and log2 fold change; The green dots represent the genes with adjusted p-value > 0.05 and log2 fold change; The blue dots represent the genes with adjusted p-value < 0.05 and log2 fold change; The gray dots represent the genes with adjusted p-value > 0.05 and log2 fold change. P+, uterine horn with the embryo; P−, uterine horn without the embryo; NB, nonbred. The pathways originated in the Ingenuity Pathway Analysis (IPA) software (Qiagen).

In a comparison between the P+ and NB groups, there were 1,101 DEGs, with 466 genes upregulated and 635 downregulated in P+ relative to NB (Fig. 2A1). The main upregulated genes were associated with pathways related to the wound-healing signaling pathway, regulation of the epithelial-mesenchymal transition by growth factors pathway, corticotropin-releasing hormone signaling, and GnRH signaling, while the downregulated genes were associated with the oxytocin signaling pathway, s100 family signaling pathway, WNT/ca + pathway, insulin secretion signaling pathway, estrogen receptor signaling, and leukocyte extravasation signaling (Fig. 2A2,A3).

A total of 1,229 DEGs were found by comparing the P− group to the NB group, with 511 upregulated and 718 downregulated (Fig. 2B1). The most significant canonical pathways associated with upregulated genes in the P− group were the antioxidant action of vitamin C, GnRH signaling, calcium signaling, prolactin signaling, and chemokine signaling, while the most significant pathways associated with downregulated genes were the S100 family signaling pathway, oxytocin signaling pathway, estrogen receptor signaling, nitric oxide signaling, leukocyte extravasation signaling, and integrin signaling (Fig. 2B2,B3).

**Figure 3:**
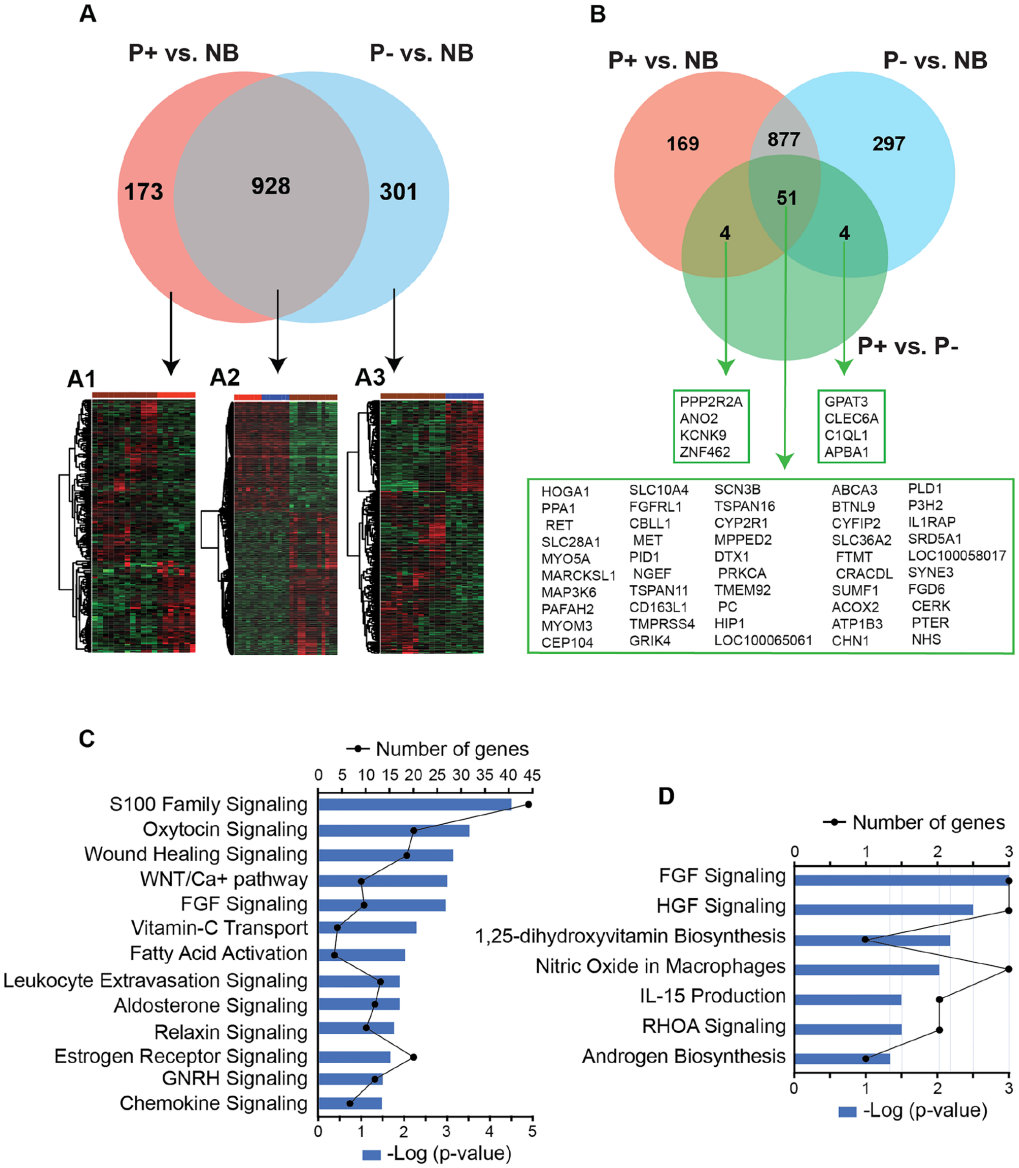
Venn diagram displaying the overlapping DEGs. (**A**) overlapping DEGs among P+ vs. NB and P− vs. NB; (**A1**) Heatmap from 173 genes only expressed P+ vs. NB; (**A2**) Heatmap from the 928 common genes between P+ vs. NB and P− vs. NB; (**A3**) Heatmap from 301 genes only expressed in P− vs. NB; (**B**) overlapping DEGs among P+ vs. NB, P− vs. NB, and P+ vs. P− [heatmaps were generated using the ggplot2 package in R]; (**C**) The most significant canonical pathways from the 928 genes common between P+ vs. NB and P− vs. NB; (**D**) The most significant canonical pathways from the 50 genes common among P+ vs. NB, P− vs. NB, and P+ vs. P−. P+, uterine horn with the embryo; P−, uterine horn without the embryo; NB, nonbred.

The overlapping genes among P+ vs. NB, P− vs. NB, and P+ vs. P− are shown (Fig. 3). There were 173 DEGs found only in P+ vs. NB, and 301 DEGs uniquely found in P− vs. NB comparison (Fig. 3A). Among the 173 genes, the upregulated genes were associated with the immune response (*C5AR2, MS4A1, FSTL1,* and *CD19*), tissue development (*LAMC3, NOX4, TNC,* and *HOX13*), p53 pathway, cell population proliferation (*PRKG1*, *PTPRZ1*, *CRLF1*), and fatty acid transmembrane transport (*THBS1*), while the downregulated genes were involved in the immune system (*FOXP3* and *IFNG*) and steroid hormone metabolism (*CYP3A95*). Among the 301 genes only found in P− vs. NB comparison (Fig. 3A), the downregulated genes were associated with the immune system (*SOCS1, CAMK2A*, and *CSF1*), IL-7 signaling (*CCND3, FOXO1, SOCS1*), potassium voltage-gated channel (*KCNH1, KCNAB3,* and *KCNG3*) and calcium voltage-gated channel (*CACNG1*). There was a total of 928 overlapping genes among the comparisons (P+ vs. NB and P− vs. NB) (Fig. 3A), showing similar expression patterns between pregnant and non-pregnant samples regardless of the embryo locations. The upregulated genes were associated with the wound-healing signaling pathway, growth factors pathway, vitamin C transport, corticotropin-releasing hormone signaling, fatty acid activation, and GnRH signaling, while the downregulated genes were associated with the oxytocin signaling pathway, s100 family signaling pathway, WNT/ca + pathway, insulin secretion signaling pathway, estrogen receptor signaling, and leukocyte extravasation signaling.

**Figure 4:**
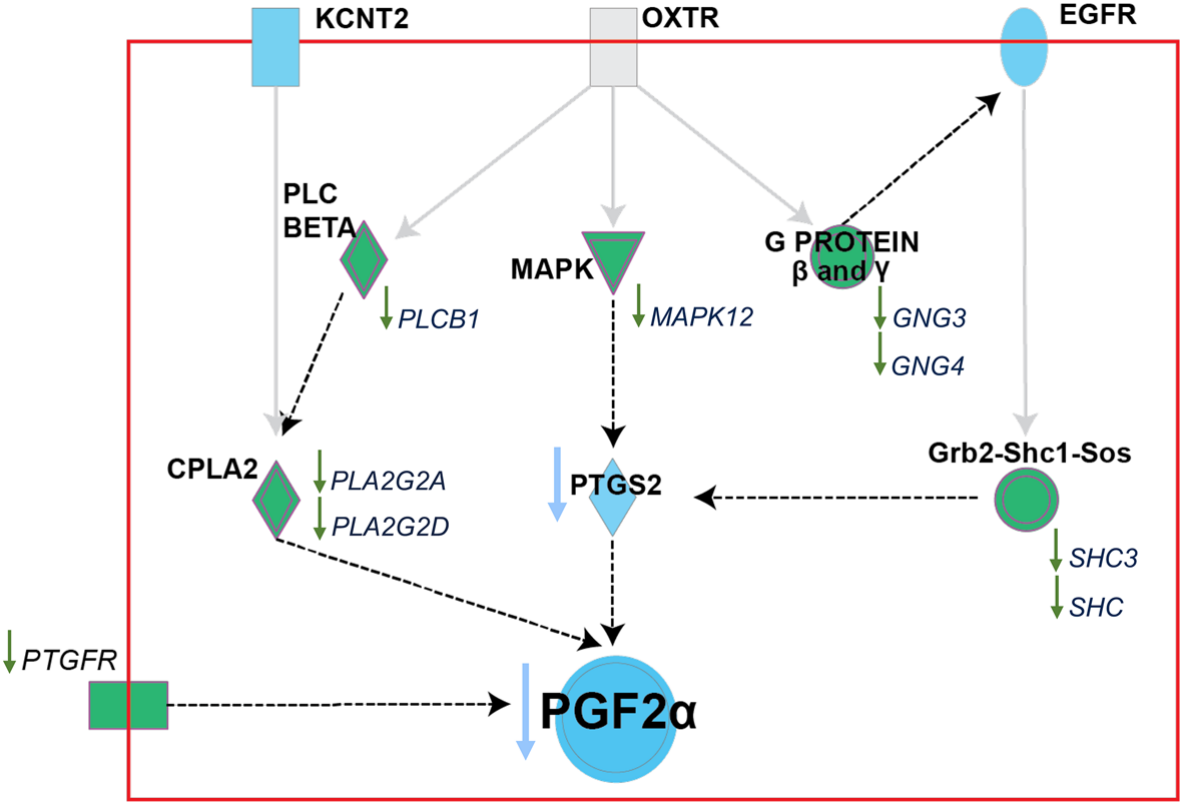
The oxytocin canonical pathway originates in the Ingenuity Pathway Analysis (IPA) software (Qiagen). The green arrows indicate the downregulation of the genes in both pregnant groups relative to the nonbred group (P+ vs. NB and P− vs. NB). The blue color indicates the prediction of downregulation of the genes (*KCNT2*, *EGFR*, and *PTGS2*) or inhibition of the hormone (PGF2α). *OXTR*, oxytocin receptor; *KCNT2,* potassium sodium-activated channel subfamily T member 2; *EGFR,* epidermal growth factor receptor; PTGFR, prostaglandin F receptor; *PLCB1,* phospholipase C beta 1; *PLA2G2A,* phospholipase A2 group IIA; *PLA2G2D,* phospholipase A2 group IID; *MAPK12*, mitogen-activated protein kinase 12; *GNG3,* G protein subunit gamma 3; *GNG4,* G protein subunit gamma; *SHC,* SHC adaptor protein; *SHC3,* adaptor protein 3; *PTGS2,* prostaglandin-endoperoxide synthase 2; PGF2α, prostaglandin F2α.

The oxytocin signaling pathway from the common 928 genes expressed between P+ vs. NB and P− vs. NB is shown (Fig. 4). There was downregulation of the prostaglandin F receptor (*PTGFR*), phospholipase C beta 1 (*PLCB1),* phospholipase A2 group IIA (*PLA2G2A)*, phospholipase A2 group IID (*PLA2G2D)*, mitogen-activated protein kinase 12 (*MAPK12)*, G protein subunit gamma 3 (*GNG3)*, G protein subunit gamma (*GNG4)*, adaptor protein 3 (*SHC3).*

To identify the molecules that could potentially control the expression of DEGs in pregnant mares in comparison to non-pregnant mares, an upstream regulator analysis was conducted. This analysis resulted in the prediction of 67 regulators in P+ vs. NB and 54 regulators in P− vs. NB (P−value of overlap < 0.05 and Z-score ≥ 2). The complete list of predicted upstream regulators for both comparisons is presented in supplementary tables 2 and 3, respectively. The upstream regulators found in P+ vs. NB, were compared with the list of expressed genes in the equine embryo (Vegas 2022) to investigate if any of the upstream regulators could have originated from the embryo (Table 1). Several genes, including *CITED2*, *GPS2*, *TP53*, *CREM*, *PDX1*, and *KDM5B* were found to be expressed in the equine embryo and could potentially lead to a change in the gene expression of the endometrium.

**Table 1:** Potential genes involved in embryo-endometrium interaction in P+ vs. NB.

Upstream Regulator*	Molecule Type	z-score	p-value	Target Molecules in Dataset**
*COP1*	Enzyme	2.98	0.0003	*CXCL10,CYP26B1,ETV4,ETV5,GPNMB,HSPB8,IL12B,PEX11A,PPARGC1A,RET*
*COL18A1*	Other	2.76	0.01	*CCND1,EPHB1,ID1,ID3,KNG1,PLAU,THBS1,TNF*
*CITED2*	Transcription regulator	2.76	0.02	*CXCL10,EGLN3,FOXP3,FUT8,GADD45B,GATA6,IL12B,KYNU,MID1,PLAU,TNF,TNFAIP3,UBD*
*GPS2*	Transcription regulator	2.75	0.002	*CCL20,CCND1,CXCL10,IL12B,PID1,RAB20,TNF,VCAM1*
*TP53*	Transcription regulator	2.58	6.4 × 10^–7^	*ABCC1,ABCG2,ACSL1,ADAMTSL4,AKT3,ALDH1A1,ALOX15,APOA1,ATF3,BHLHE40,BOK,CAMK2N1,CAV1,CCN2,CCND1,CDC42EP3,CENPK,COL6A2,COQ8A,CRYAB,CXCL10,CYFIP2,CYP24A1,CYP26B1,DAPK1,DHCR24,DKK3,DLX1,DNMT3A,DPYSL4,DTX1,DUSP4,DUSP5,DUSP6,EGF,EGR2,FGF7,FHL1,FHL2,FKBP1B,FOXP3,FRMD4A,FSTL1,GADD45B,GATA6,GJB3,GNAI1,HS3ST1,ID1,ID2,ID3,IFNG,IGDCC4,IGFBP3,IGFBP7,IL31RA,IRS1,ITGA2,ITGB6,LOX,LRAT,LTBP1,MAN2A1,MAPK12,MET,MIS18BP1,MYBL2,MYO10,MYO5A,NOS3,NOX4,NPNT,NPTX1,NR4A3,OAT,PBK,PCK1,PHGDH,PHLDA1,PLAU,PPARGC1A,PPIC,PPP1R13B,PRKCA,PRKG1,RGS16,ROBO1,S100A4,SALL2,SCD,SCN3B,SCTR,SHROOM3,SLC2A1,SPHK1,STEAP3,THBS1,TIMP1,TMEM43,TMSB10/TMSB4X,TNC,TNF,TNFAIP2,TNFRSF11A,TNFRSF18,TUBB3,TUBB4A,UBD,UBE2T,UGDH,VIM,VLDLR,WNT5B*
*NR3C1*	Ligand-dependent nuclear receptor	2.49	4.9 × 10^–9^	*ABL1,ACTN2,AQP1,BHLHE40,BOK,CAV1,CCL20,CCL22,CCN2,CCND1,CDC42EP3,CIDEC,COL6A2,COQ8A,CRYAB,CSRNP1,CXCL10,DDC,DUSP4,ERRFI1,GADD45B,GLCCI1,GRIA2,HGD,HSD11B1,HSD11B2,IFNG,IGFBP1,IL12B,IL1RAP,ITGB6,LOX,MFSD2A,NFIL3,NOS3,OAT,PC,PCK1,PHLDA2,PLAGL1,PLAT,RAPGEF4,RELT,RGS2,S100G,SDS,SERPINA6,SERTAD2,SLC38A1,SLC43A1,SOX4,STC1,THBS1,TIMP1,TNF,TNFAIP2,TNFAIP3,VCAM1,VIM,WNT5A*
*CREM*	Transcription regulator	2.44	0.0001	*ATF3,BHLHE40,CCND1,CSRNP1,DUSP4,EGR2,ERRFI1,GADD45B,IFNG,NFIL3,NFKBID,NR4A1,NR4A2,PCSK1,THBS1,TNF*
*PDX1*	Transcription regulator	2.38	3.6 × 10^–7^	*ACE2,ATF3,CAMK2N1,CCND1,DUSP5,DUSP6,EPHB1,GCH1,HAP1,ID1,ID2,ID3,IGFBP3,NPTX2,PC,PCSK1,PHGDH,PRSS23,S100G,SLC2A1,ST18,TSPAN8,WFS1*
*KDM5B*	Transcription regulator	2.34	0.002	*CAV1,CCN2,CCND1,DHCR24,FHL1,FJX1,ID2,PBK,PDE3B,PIR,SOX4,TUBB2A,WNT7B*
*INSIG1*	other	2.33	0.01	*ATF3,CCL22,CXCL10,DHCR24,IFNG,IL12B,LIPA,PLD1,SCD*
*SPRY2*	Other	2.28	0.00002	*CAV1,CCL20,CXCL10,DUSP6,ETV4,ETV5,ID2,IFNG,IGFBP3,ITGA2,MET,MYCN,SATB1,SLC2A1*
*PRKAA1*	Kinase	2.24	4.8 × 10^–9^	*ATF3,CA2,CDC42EP3,EGF,EGLN3,HSD3B2,ID2,IFNG,IL12B,KLHL29,MMP16,OAT,PARP3,PLA2G2A,PPARGC1A,RGS2,RHOU,RTN4RL1,SLC2A1,SPRED2,THRSP,TNF,TNS1,VCAM1,ZNF462*
*SAV1*	Other	2.23	0.001	*CCN2,CCND1,SOX4,TNF,VIM*
*DNASE2*	Enzyme	2.21	0.004	*ACKR4,CXCL10,GPNMB,IFNG,TNF*
*SERPINF1*	Other	2.2	0.004	*CCN2,CCND1,CRYAB,CSPG4,PPARGC1A,THBS1,VIM*
*TNFAIP3*	Enzyme	2.17	0.01	*CCL20,CXCL10,IFNG,TNF,TNFAIP3,VCAM1*
*TIA1*	Other	2.13	0.007	*AGR2,CCN2,CD109,GATA6,PPARGC1A,S100A6,TIMP1,TNF*
*DICER1*	Enzyme	2.06	0.001	*CCND1,CXCL10,DEPP1,DHCR24,EGR2,FOXP3,ID3,IFNG,ITGA2,KRAS,NOS3,PLAU,PRKCA,RET,RGCC,RGS5,SPRY2,THBS1,TNF,VIM,WNT5A,WNT7B*
*CD38*	Enzyme	2.00	0.03	*CCL22,EGLN3,LRRC8C,NFIL3,NOX4,PPARGC1A,S100A4,S100A6,SLC2A1,VCAM1,VIM*

*Upstream regulators: Common upstream regulators found in this study and were also expressed in the D12 embryo in a previous study (Vegas 2022). These upstream regulators could be potentially originate from the mobile embryo and trigger changes genes in the endometrium (target genes).

**Target molecules in dataset: Genes found in this study that are potentially affected by the upstream regulators.

### Dynamics of gene expression between the horn with and without embryo in the pregnant group

There were 59 DEGs when comparing P+ vs. P−, including 30 upregulated and 29 downregulated genes in P+ related to P−. The upregulated genes were involved in the regulation of vascular endothelial growth factor expression (*MAP3K6*), wound healing signaling (*IL1RAP*), insulin secretion (*PC*), angiogenesis (*PLD1*), nutrient transporter (*SLC36A2*), and degradation of fatty acids (*ACOX2*), while the main downregulated genes were involved with triacylglycerol biosynthesis (*GPAT3*), immune system (*IFNG*), relaxin signaling (*MPPED2*), synthesis of cholesterol and lipids (*CYP2R1*), and potassium channel (*KCNK9).*

There were 51 overlapping genes among P+ vs. NB, P− vs. NB, and P+ vs. P− (Fig. 3B). The upregulated genes were involved in the regulation of vascular endothelial growth factor expression (*MAP3K6*), wound healing signaling (*IL1RAP*), insulin secretion (*PC*), angiogenesis (*PLD1*), and nutrient transporter (*SLC36A2*), while the main downregulated genes were involved with relaxin signaling (*MPPED2*), and synthesis of cholesterol and lipids (*CYP2R1*).

## Discussion

The endometrium serves as the crucial interface between the maternal body and the embryo, and its receptivity and responses can significantly influence pregnancy outcomes. Understanding the interactions between the embryo and endometrium is vital to gain insights into the causes of embryonic loss and potential methods of improving equine fertility. In this context, our study takes a critical step forward, shedding light on the embryo-endometrial interaction associated with the location of the embryo during the mobility phase in mares. To our knowledge, this is the first study that characterized the global transcriptome profile and compared gene expression from the endometrium of pregnant versus nonpregnant mares on day 12 after ovulation, considering the location of the embryo in pregnant mares.

In this study, the endometrial samples were collected using a cytobrush. Different studies in cattle^29,30^ and in horses^17,31–33^ have demonstrated that cytobrush sampling is a non-invasive method for obtaining RNA of a suitable quantity and quality for gene expression analysis from the endometrium. Moreover, the transcriptome of the endometrial luminal epithelium (LE) corresponds to the major gene expression observed between pregnant and nonbred mares^23,25^. Here, we collected endometrial cells corresponding to the LE, which reflects the primary endometrial response to signaling molecules emitted by the conceptus during the mobility phase in mares^25^. Using the cytobrush also enabled us to obtain samples in particular areas of the uterus and close to the mobile embryo, allowing us to detail the embryo-endometrial interaction.

The role of the immune system during the early stages of pregnancy is crucial for establishing a balanced environment during early pregnancy, ensuring protection for both the mother and the developing embryo^34^. This finely tuned balance is critical for the support of the pregnancy. Chemokines, constituting a large group of chemotactic cytokines, have established roles in recruiting and activating leucocytes^35^. In our study, we observed a downregulation of four chemokines (*CCL20*, *CCL22*, *CCL2*, and *CXCL10*) in pregnant mares (P+ and P−) compared to the nonbred (NB) group; these chemokines are typically involved in the inflammatory response^35^. Notably, among these, *CXCL10* has been shown in previous research to be associated with the T lymphocyte homing^36^, highlighting its crucial role in immune regulation. Another upregulated gene in this study, the glycoprotein *CD55* that possesses immunosuppressive qualities, was highlighted in the literature^37^. Particularly, our findings align with previous research, which reported downregulation of the inflammatory response in the luminal epithelium (LE)^23^. As such, our results underline the necessity of precise immune system regulation in the LE during early pregnancy stages.

In the present study, several genes linked to vital processes at the embryo-maternal interface were found to be upregulated in pregnant mares (P+ and P−) compared to the NB group, including the fibroblast growth factor 9 (*FGF9*) known for stimulating endometrial proliferation^27^. Another gene was the TIMP metallopeptidase inhibitor 1 (*TIMP1*), which is proposed to be associated with endometrial tissue remodeling^38^. In the human endometrium *TIMP1* is essential during implantation for promoting trophoblast invasiveness. Our findings also highlighted two key genes that play a critical role in nutrient delivery, particularly the transport of amino acids and fatty acids: the lipid carrier ganglioside GM2 activator (*GM2A*)^39^ and a member of the solute carrier family 36, *SLC36A2*^40^. Two other notable genes that were upregulated in the pregnant groups, heat shock protein family B (small) member 8 (*HSPB8*) and crystallin alpha B (*CRYAB*), are known to be regulated by estrogens and have roles in enhancing endometrial receptivity to implantation in humans^41^. Additionally, the solute carrier family 2 member 1 (*SLC2A1*) that was upregulated in our study is the predominant glucose transporter in human and mouse endometrium, where it is essential for decidualization^42^. In horses, *SLC2A1* appears to be stimulated by the early conceptus and serves as the primary contributor to uterine luminal fluid during early pregnancy^43^. Epidermal growth factor (*EGF*) was also upregulated in pregnant mares (P+ and P−) compared to the NB group, which implies its crucial role in the instigation and evolution of both maternal and fetal interface tissues over the course of gestation^44^. Our findings thus underline the complexity and importance of transcriptomic regulation at the maternal–fetal interface.

Two members of the insulin-like growth factor system (*IGFBP1* and *IGFBP3*) were upregulated in pregnant mares compared to the NB group in our study. The *IGFBP1* has been assigned to play a critical role in preparing the endometrium for successful implantation, as well as supporting embryonic and placental growth and development^45,46^. Further, it has been suggested that *IGFBP1* in ruminants is stimulated by interferon-tau, P4, and prostaglandins, and plays a pivotal role in facilitating communication between the conceptus and endometrium^45^. Similarly, *IGFBP3* is observed in pregnant mares and is considered instrumental in the intricate dialogue between the endometrium and conceptus during the early stages of pregnancy and preparation for implantation^24^. These findings accentuate the potential importance of the *IGFBP*s, specifically *IGFBP1* and *IGFBP3*, in facilitating the successful progression of pregnancy in mares.

During early pregnancy, angiogenesis plays a pivotal role in enhancing blood flow to the uterine tissue, a process crucial for efficient nutrient transport and embryonic nourishment^47^. In our study, upregulated genes in pregnant mares (P+ and P−) compared to the NB group, involve WNT family member 7B (*WNT7B*), nuclear receptor subfamily 4 group A member 1 (*NR4A1*), and fibroblast growth factor 9 (*FGF9*). *WNT7B*, was previously found during early pregnancy in mares^48^, and is believed to contribute to the formation of capillary lumens in the renal medulla^49^. Similarly, *NR4A1* has been linked with vascular permeability in mouse studies^50^ and *FGF9* indirectly influences vascular development by promoting smooth muscle cell growth and micro-vessel formation^51^. The upregulation of *FGF9* on D12 in pregnant mares was linked to the estradiol (E2) secreted by the embryo^21^. Furthermore, *FGF9* is recognized as an embryonic growth factor in pigs^52^, and its induction by prostaglandin E2 (*PGE2*) has been observed in the human endometrium^53^. Our findings align with previous studies involving pregnant and non-bred mares, where DEGs in the LE were not typically associated with classical angiogenic markers compared with the results from a complete biopsy sampling^23^. Moreover, it has been suggested that on D12 of pregnancy in mares there is a remodeling of vascularization rather than neo-angiogenesis^21^.

In the present study, downregulation of phospholipase A2 group IIA (*PLA2G2A*, *PLA2G2C*, and *PLA2G2D*), phospholipase C (*PLC*), and prostaglandin F2alpha receptor (*PTGFR*), and upregulation of the nuclear factor interleukin 3 regulated (*NFIL3*) in pregnant mares (P+ and P−) compared to the NB group suggests a downregulation of oxytocin pathway in order to inhibit PGF2α synthesis. In mares, it has been demonstrated that oxytocin stimulates PGF2α synthesis^54^. Also, an in vitro study demonstrated that COX-2 is involved in the mechanism by which oxytocin regulates PGF2α production in the endometrial cell in mares^55^. Oxytocin binds to a cell-surface membrane receptor and activates a complex intracellular signaling pathway which ultimately leads to the activation of PLA2^56^. In the endometrium, PGF2α synthesis is regulated by PLA2 by regulating the release of arachidonic acid^57^. In mares, *PLA2G2A* increased after a decrease of P4^58^, and PLA2 activity has been correlated with the period of maximum PGF2α secretion during luteolysis^59^, which suggests the involvement of PLA2 in the synthesis of PGF2α. Similarly, PLC has been suggested to be involved in PGF2α synthesis through the effect of oxytocin^60^. *NFIL3* has been demonstrated to be essential for the development of a subset of uterine natural killer cells secretion growth-promoting factor in both humans and mice^61^ and to inhibit the induction of prostaglandin-endoperoxide synthase (*PTGS2*)^62^. These findings contribute to our understanding of how oxytocin and other components may contribute to the regulation of PGF2α production and endometrial cell mechanisms in mares. The endometrial gene expression of *PTGFR* during early pregnancy in mares has had some controversy in the literature, while *PTGFR* inhibition has been described on D14^63^, it was not differentially expressed on D12^23^ or upregulated during early pregnancy^25^. The luteolytic period in mares varies among individuals^64^ and the downregulation of *PGFR* in pregnant mares (P+ and P−) compared to NB group in our study could be attributed to the animals used being close to luteolysis (at least one day before) or to the different methods of sample collection, cytobrush vs. biopsy. Moreover, an endometrial auto-amplification system in which PGF2α can stimulate its own production has been proposed in mares^33,65^, which would align with the downregulation of *PTGFR* in this study. Another downregulated gene in pregnant mares (P+ and P−) in our study, tumor necrosis factor (*TNF*), has been reported to increase PGF2α secretion in CL^66^ and endometrium in mares^67^, and could be involved in the prevention of luteolysis in mares. Future research should investigate the role and regulation of PGF2α secretion during early pregnancy in mares, especially on genes downstream of OXTR that could be important in understanding the maternal recognition of pregnancy in mares.

The lack of a distinct increase in the classical machinery for synthesis, transport, and metabolism (*OXTR, COX-2, PTGFS, SLCO2A1,* and *HPGD*) of PGF2α indicates that the nonpregnant mares in the study had not started luteolysis. This is further supported by findings from our previous study in which concentrations of P4 from the same breed of horse were not different between pregnant and nonbred mares until the beginning of luteolysis on mean D13^68^.

In this study, the IPA analysis revealed that several genes could be influenced by the predicted upstream regulators, a pattern that aligns with the expression profiles of these regulators in a prior study on embryos^28^. Several genes associated with immune system regulation, including *IFNG, CCL20, CXCL10, TNF, TNFAIP3*, and *VCAM1*, were observed to be affected by the upstream regulators. Interferon-gamma (*IFNG*), a pro-inflammatory cytokine, holds a pivotal position in the realm of allogeneic cell applications due to its innate and adaptive immune response activation properties^69^. The regulatory functionality of tumor necrosis factor (*TN*F) extends to guiding the shift from an antigen-responsive to an inflammatory state during terminal T cell differentiation^70^. The protein induced by tumor necrosis factor-alpha (*TNFAIP3*) is postulated to orchestrate inflammation regulation through T helper cell differentiation and associated cytokines^71^. Additionally, the vascular cell adhesion molecule (*VCAM1*), an immunoglobulin gene superfamily member^72^, has been observed in bovine embryos and endometrium, with a presumptive role in establishing conceptus adhesion. Given these findings, it becomes imperative to probe further into the role of upstream regulation, specifically its influence on maternal-side actions. Subsequent research should aim to elucidate these mechanisms, thereby paving the way for a comprehensive understanding of immune system regulation in pregnancy.

The equine embryo can be visualized through ultrasonographic images on Days 9 to 10 when is found to be mobile in the uterus^6^. During these days, the number of transuterine migrations is minimal, and more than 50% of the total time is spent in the uterine body. From Day 11 until around Day 16, before fixation occurs, the mobility of the embryo reaches its peak and the embryos spend more time in the uterine horns^73^. In our study, it was observed that during the cytobrush collection in P+, the embryos had already left the P− at least 50 min before the sample collection. This finding is consistent with previous studies that suggest on Day 12, the duration of movements from one horn to another may take anywhere from 67 to 120 min^18,73,74^. Additionally, equine embryo mobility does not follow one direction and the embryo can move either cranially or caudally from its position^75^.

In the present study, we observed that there were no differences in gene expression between the uterine horns on the same side and the opposite side of ovulation in nonbred mares. This indicates that the CL does not affect the gene expression in the uterus. However, due to sample size restrictions, we were unable to fully evaluate the effect of the CL side on the transcriptome of the endometrium in the horn with and without an embryo.

Lastly, our hypothesis that the presence of a mobile embryo induces local changes in the gene expression of the endometrium was supported. A total of 30 genes were upregulated, and 29 genes were downregulated in the pregnant horns with an embryo (P +) compared to the pregnant horns without an embryo (P−) after the embryo had been located for at least 10 min. A study in humans classified genes based on their length, with genes with an average transcript length of 7,878 categorized as small, taking ~ 6 min to be fully expressed while genes with an average transcript length of 50,753 classified as medium, taking ~ 30 min to be expressed, and genes with an average transcript length of 133,142 categorized as large, taking ~ 60 min to be expressed^76^. In our study, all DEGs between P+ and P− are classified as small transcripts, with an average length of 3,244.5 ± 295.7. These DEGs have the potential to be transcribed in approximately six minutes, based on a previous study^76^. However, to our knowledge, the exact time genes are fully transcribed after a stimulus in horses is unknown.

Among the upregulated genes, the interleukin 1 receptor accessory protein (*IL1RAP*), has been previously reported in the embryo as well as in the endometrium of pregnant mares^77^. In pigs, *IL1RAP* was highly expressed in the LE during the period coinciding with maternal recognition of pregnancy, on D12^78^. In this species, it has been hypothesized that the expression of *IL1RAP* is regulated by estrogen^79^. Hence, the observed upregulation of *IL1RAP* in the horn possessing the embryo in our study could be attributed to the E2 known to be secreted by the equine embryo. In addition, *IL1RAP* is essential for activating *IL1B*, which consequently augments the expression of *PTGS2* and *PTGES* in the endometrium during the attachment period in pigs^80^. While no difference was found in *PTGES* in this study, the increase of *IL1RAP* could be associated with *PTGE* production*,* as mentioned in the previous study in pigs. Treatment with prostaglandin E2 has been shown to stimulate myometrial contractions^81^, and it could be associated with a stimulus of embryo mobility. The upregulation of *KCNK9*, a gene associated with potassium channel activity, also may play a role in embryo mobility, given that potassium channels have been linked to uterine contractility^82^. Another upregulated gene in P+ in comparison to P−, acyl-CoA oxidase 2 (*ACOX2*), is an enzyme involved in the peroxisomal beta-oxidation of fatty acids, particularly branched-chain fatty acids, and bile acid intermediates^83^. In this regard, intrauterine administration of plant oils, a source of fatty acid, during diestrus in mares, caused maintenance of the CL^84^. A study using bovine endometrial cells indicated that the production of prostaglandins can be modulated in both the quantities and types of prostaglandins and the ratio of PGF to PGE, depending on the type and ratio of fatty acids^85^. Another interesting upregulated gene in P+ is the solute carrier family 36 member 2 (*SLC36A2)*, which mediates the transport of amino and fatty acids^40^. The mitogen-activated protein kinase kinase kinase 6 (*MAP3K6*) mediates angiogenic and tumorigenic effects via vascular endothelial growth factor expression^86^. The phospholipase D1 (*PLD1*) is an enzyme involved in several cellular processes important for cell survival, such as G protein and tyrosine kinase receptor signaling pathways, membrane trafficking, and regulation of cell cycle control^87^. Therefore, the upregulation of genes mentioned above could potentially be regulated locally by the embryo and play an important role in early pregnancy in mares. Given that the size of a transcript can significantly impact gene transcription in response to a stimulus^76^, and considering the limited information available on the time it takes for genes to be expressed in equines after a stimulus, a possibility that the P− could exhibit distinct changes in response to the embryo cannot be eliminated. This variation could be explained by a prolonged duration of transcription for some genes in P−. According to a previous report that categorized the time taken for a gene to be expressed based on the size of the transcript^76^, all differentially expressed genes between P+ and P− in our study could be classified as small transcripts, speculating that the presence of the embryo for at least 10 min in a specific location of the uterus should provide sufficient time for the expression of the genes that were identified. Future studies are needed to characterize the time required for genes to be fully transcribed after a stimulus in horses.

Although our current study focuses on transcriptomic changes, we acknowledge the importance of the non-genomic mechanisms, such as rapid signaling cascades and activation of intracellular pathways that operate independently of transcriptional modifications. Future investigations integrating both genomic and non-genomic responses would provide a more holistic understanding of the intricate and multifaceted nature of embryo-maternal interactions, particularly during the critical period of embryo mobility and early implantation in mares.

### Conclusion

The present study provides valuable insights into the molecular mechanisms and pathways involved in early pregnancy and suggests a local effect of the embryo that could have important relevance in embryo-maternal communication during early pregnancy in mares. This study advances our understanding of equine reproductive biology and serves as a model for future studies involving local embryo-maternal interaction.

## Material and methods

All experimental procedures were conducted from January to March in southeastern Brazil (latitude, 23°; longitude, 44°). All the experimental protocols were approved by the Committee on Ethics in the Use of Animals (CEUA) in accordance with CEUA protocol number 0022–20-2018. All methods are reported in accordance with ARRIVE guidelines. The mares were maintained on pasture under natural light and received trace-mineral salt with free access to water. Sixteen multiparous Mangalarga Marchador non-lactating mares aged from 6 to 13 years weighing 382 to 478 kg were enrolled in the study. Abnormalities of the reproductive tract including fluid during diestrus were not detected by transrectal ultrasonic imaging in the enrolled animals^88^. All mares remained healthy and in good body condition throughout the study.

### Insemination and experimental design

Mares with a mature CL were treated intramuscularly with a luteolytic dose (5 mg) of PGF2α (dinoprost tromethamie, Lutalyse, Zoetis, SP, Brazil), and the ovaries were scanned daily until detection of a ≥ 35 mm preovulatory follicle and an endometrium echotexture characteristic of estrus^89^. Artificial insemination was done 24 h after induction of ovulation with 1000 IU of hCG (Vecor, Hertape Calier®). Fresh semen from two fertile stallions was diluted with a semen extender (Botusemen®; Botupharma, Brazil) and an insemination dose containing 20–30 mL (500 × 10^6^ cells/mL with progressively motile sperm) was used. Ovulation was determined daily between 8 and 9 AM, and the day of ovulation detection was designated as D0. Pregnancy diagnosis was done by ultrasonography on D12 using duplex B-mode (gray scale) and color Doppler ultrasound machine (Mindray Z5-Vet; Mindray North America, Mahwah, NJ, USA) with a linear-array 7.5-MHz transrectal transducer.

A total of 10 mares were bred to obtain the 8 pregnancies. This study includes pregnant mares (n = 8) and nonbred mares (n = 6) that were not inseminated. The CL echogenicity, CL area, and cross-sectional area of CL with blood flow were evaluated as described^90^ to confirm that luteolysis had not begun. On D12, each mare in the nonbred and pregnancy groups had CL blood flow ≥ 60% of CL cross-sessional area and CL diameter (≥ 5.8 cm^2^) consistent with a mature functional CL^90^.

### Sample collection

For the collection of endometrial samples, the uterus was partitioned into three theoretical segments of similar length for each uterine horn and the uterine body, as previously described^91^. Briefly, the uterus was divided into caudal segment of the uterine body, middle segment of the uterine body, cranial segment of the uterine body, caudal segment of the right horn, middle segment of the right horn, cranial segment of the right horn, caudal segment of the left horn, middle segment of the left horn, and cranial segment of the left horn. On D12 in the pregnant group, endometrial samples were collected from the middle segment of the uterine horn with (P +) and without the embryo (P−) using a cytobrush (PROVAR®—São Paulo, Brazil). The transrectal ultrasonic imaging was done every 10 min until the embryonic vesicle had moved from the cranial segment of the uterine body to the middle segment of one of the uterine horns. Once the embryo was located in the middle segment of the uterine horn for at least 10 min, the cytobrush was inserted transvaginally and gently introduced into the uterine horn that contained the embryonic vesicle. The cytobrush was guided by ultrasonography and the endometrial samples were collected in the uterine horn approximately 5 mm caudal to the embryo. The cytobrush was rotated for ~ 20 s to retrieve the endometrial cells^29,30^. At least 50 min had passed since the embryo left the P− in all the mares before the cytobrush sample collection in the P+.”

Immediately after the sample collection from the uterine horn with the embryo, a second endometrial cytobrush sample was collected in the middle segment of the opposite uterine horn that did not contain the embryo. After collecting a sample from the P−, the embryo was visualized using transrectal ultrasound to ensure it was undamaged and still present in the P+. In the nonbred group on D12, endometrial cytobrush samples were collected in the middle of the left and right horns. After sample collection, the cytobrush was immediately placed into a 4.5 mL cryogenic storage tube containing 1.5 mL of RNA*later* (Thermo Fisher Scientific, Waltham, MA, USA), kept 24 h at –20 °C, and then stored at –80 °C.

### RNA extraction, library preparation, and sequencing

The RNA from a total of 26 endometrial samples was extracted using RNeasy Micro Kit (Qiagen) per manufacturer’s instructions. The RNA quantity was measured by spectrometry with a NanoDrop 2000 spectrophotometer (Thermo Scientific). Library preparation from the total RNA samples was performed using the TruSeq Stranded mRNA Sample Prep Kit (Illumina, San Diego, CA, USA) to purify mRNA from total RNA using poly-dT beads per manufacturer’s instructions. The libraries were quantitated by qPCR and sequenced for 151 cycles from each end of the fragments on a HiSeq 4000 platform using a HiSeq 4000 sequencing kit version 1 according to the manufacturer’s instructions, generating 9 Gb reads, 150PE, stranded, per sample (IDseq Inc.).

### Statistics and data analysis

The raw reads were trimmed using TrimGalore v0.4.4 (Babraham Bioinformatics) with the quality threshold set to a minimum Phred score of 30. Trimmed reads were aligned to the current equine reference genome (EquCab3.0; database release 104) using STAR v2.7.2a and quantified using featureCount (V 1.6.2)^92^. K-Means clustering analysis was performed among the four main clusters of the most variable genes, based on the standard deviation of gene expression throughout all samples. This was followed by an enrichment analysis that outlined the key pathways linked to each cluster^93^. The differentially expressed genes (DEGs) were identified using the DESeq2 package in R with adjusted- P−value < 0.05 and minimum fold change of 2. First, the genes from the horn with the embryo (P +) and the horn without the embryo (P−) in the pregnant mares were each compared to the non-bred horns (NB), using a nonpaired method. Next, to analyze the genes which might be locally affected by the embryo, the identified DEGs in the mentioned comparisons (P+ vs. NB, and P− vs. NB) were compared between the horn with and without embryo (P+ vs. P−) using a paired comparison. Additionally, the gene expression in the uterine horn ipsilateral to and contralateral to the corpus luteum was compared in the NB group. Heatmaps were created in the ggplot2 package in R, using the read count value and data was normalized using EdgeR log2 (CPM + c). For the PCA plot, ggplot2 was used, and read counts were normalized using the log2 (CM P+ c) transformation and defined a minimal count per million (CMP) of 0.5.

The Ingenuity Pathway Analysis (IPA) software (Qiagen) was employed to identify canonical pathways and upstream regulatory elements^26^. The Z-scores were used to predict the activation state (activation or inhibition) of each upstream regulator. The predicted upstream regulators were considered significant if they had a significant (P < 0.05) activation Z-score > 2 (activated) or < – 2 (inhibited).

The predicted upstream regulators found in our study were then compared with the previously published data of the equine embryo transcriptome to investigate the possibility of an embryo-maternal interaction^25^.

### Supplementary Information

Supplementary Information 1.

Supplementary Information 2.

Supplementary Information 3.

## Data Availability

The RNA-seq data from this study is accessible at the Sequence Read Archive database with Accession Number: PRJNA984326.
